# A Biopsychosocial Framework to Guide Interdisciplinary Research on Biathlon Performance

**DOI:** 10.3389/fpsyg.2021.671901

**Published:** 2021-04-29

**Authors:** Amelie Heinrich, Oliver Stoll, Rouwen Cañal-Bruland

**Affiliations:** ^1^Department of Sport Psychology, Sport Pedagogy and Sport Sociology, Institute of Sport Science, Martin Luther University Halle-Wittenberg, Halle, Germany; ^2^Department for the Psychology of Human Movement and Sport, Institute of Sport Science, Friedrich Schiller University Jena, Jena, Germany

**Keywords:** performance, expertise, biathlon, cross-country skiing, rifle shooting, interdisciplinary, social context, big data

Biathlon is a unique combination of two challenging and remarkably different tasks: cross-country skiing in free technique and rifle shooting in either prone or standing position. Over the past few decades, a growing body of biathlon-specific research considerably improved our understanding of the factors determining biathlon performance (for a review, see Laaksonen et al., 2018). This includes biological aspects of biathlon performance, comprising physiological parameters (e.g., Rundell and Bacharach, 1995; Stoeggl et al., 2015; Laaksonen et al., 2020) as well as biomechanical and motor control factors such as postural control, rifle stability, shoulder force, and triggering or aiming strategies (e.g., Groslambert et al., 1999; Baca and Kornfeind, 2012; Sattlecker et al., 2014; Köykkä et al., 2020). In addition, another branch of research focuses on psychological factors that influence performance, including the role of attentional processes (e.g., Gallicchio et al., 2016; Luchsinger et al., 2016; Heinrich et al., 2020), dealing with psychological pressure (e.g., Vickers and Williams, 2007; Lindner, 2017) and the effectiveness of psychological interventions (e.g., Groslambert et al., 2003; Laaksonen et al., 2011). However, with only one exception (Harb-Wu and Krumer, 2019), biathlon-specific research has largely overlooked the degree to which social context factors may impact biathlon performance. Here, we advocate a holistic approach to gain a more complete understanding of the factors contributing to biathlon performance. Admitting to the fact that biological determinants, psychological factors, and social context never occur in isolation, but instead need to be considered in conjunction, we propose a biopsychosocial framework to guide future research efforts into biathlon performance. This integrative, interdisciplinary, and holistic approach to examine biathlon performance is illustrated in Figure 1.

**Figure 1:**
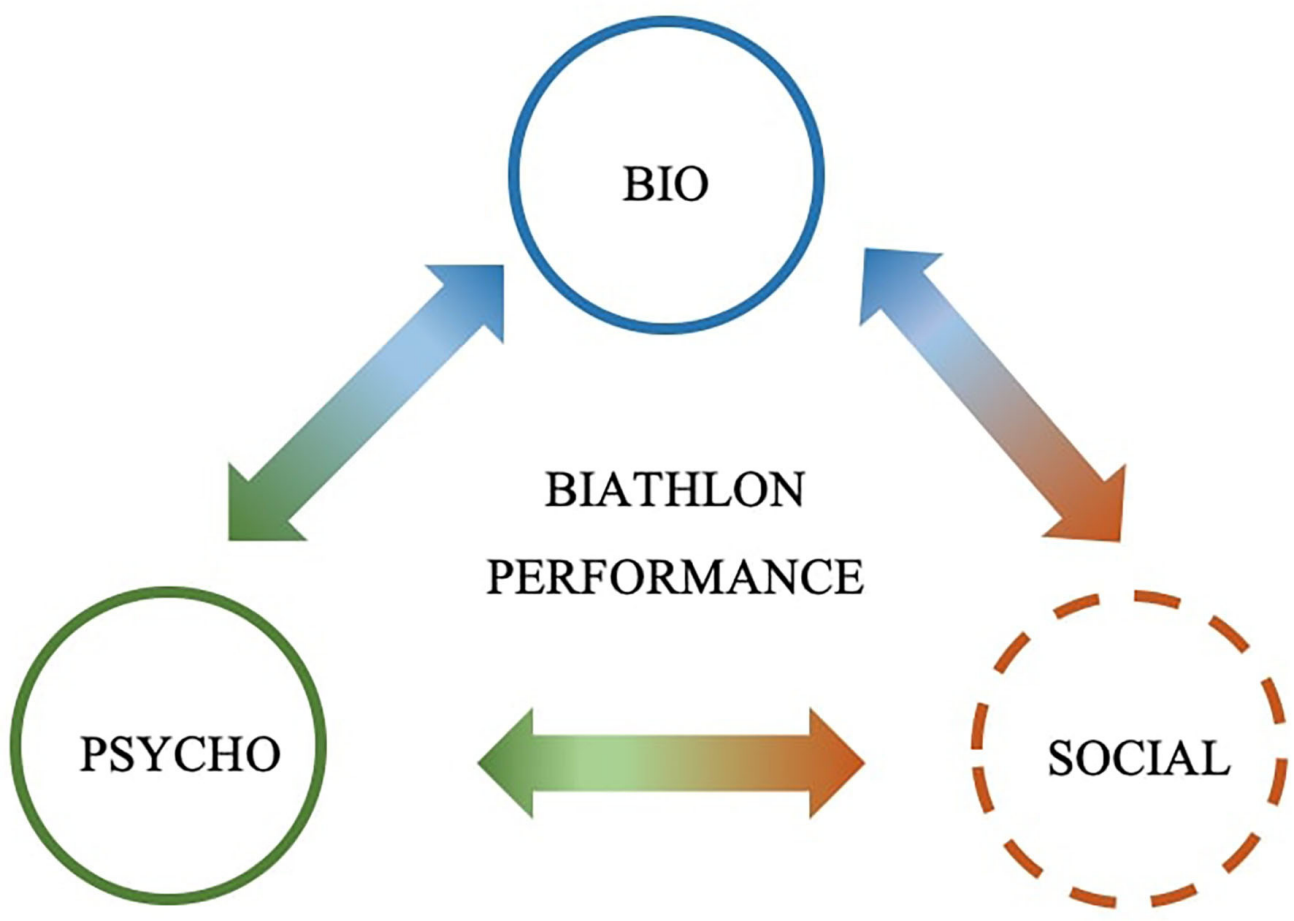
A biopsychosocial framework of biathlon performance.

Originally, biopsychosocial approaches were developed in the area of medicine and psychiatry to address limitations of the traditional biomedical model and generally aim at considering behavioral, psychological, and social dimensions when trying to understand a person's condition (Engel, 1977, 1997). Nowadays, biopsychosocial models have stepped out of their original scope and are widely used, for instance, to explain arousal regulation (Blascovich and Tomaka, 1996) or to examine stress in adolescence (Rith-Najarian et al., 2014). The application of biopsychosocial models in sport is scarce and mainly limited to the field of injuries or pain (e.g., von Rosen et al., 2017; Bumann et al., 2020). Before outlining the specific steps that need to be taken to realize a research agenda in biathlon guided by the proposed biopsychosocial framework, we shortly summarize evidence stemming from research focusing on isolated, that is, biological, psychological, and social aspects of the framework.

## In a Nutshell: Research on Biological Factors of Biathlon Performance

As concerns biological factors, research revealed, for instance, that biathletes with a larger capacity for oxygen uptake (i.e., high peak of oxygen uptake) show faster skiing times (Rundell, 1995; Rundell and Bacharach, 1995). Oxygen uptake at a lactate threshold of 4 mmol/L and gross efficiency may predict high proportions of variance in biathlon competition performance (i.e., the higher, the better; Laaksonen et al., 2020). The capacity for oxygen uptake becomes even more important as rifle carriage in skiing results in higher physiological demands such as increased oxygen costs, greater ventilation, and higher lactate values (Frederick, 1987; Rundell and Szmedra, 1998; Jonsson Kårström et al., 2019), in biomechanical adaptations (e.g., higher cycle rate and leg forces; Stoeggl et al., 2015), and in decreased performance in maximal roller skiing compared with roller skiing without a rifle (Jonsson Kårström et al., 2019). Additionally, several motor control parameters (i.e., biomechanical aspects) were shown to be reliable predictors for distinguishing between expert and less skilled biathletes: Expert biathletes are characterized by higher rifle stability (i.e., less rifle sway; Hoffman et al., 1992; Sattlecker et al., 2014, 2017) and show a more stable aiming pattern (Baca and Kornfeind, 2012) as well as higher postural control (Groslambert et al., 1999; Sattlecker et al., 2014, 2017). Furthermore, successful biathlon shooters exhibit higher force values of the rifle stock in the back shoulder and specific triggering patterns characterized by an increasing force followed by a plateau before firing a shot (Sattlecker et al., 2017; Hansen et al., 2019). Successful shots are further characterized by being fired at a specific phase of the cardiac cycle (under exercise conditions less frequently from 100 to 200 ms after the R-wave; Gallicchio et al., 2019). Dependent on the aiming strategy (the so-called *hold* vs. *timing* strategy), shooting accuracy is associated with more stable aiming at the center of the target and a decrease in total velocity of the rifle just before firing the shot (Köykkä et al., 2020). Finally, some research focused on the impact of physiological workload on shooting performance, revealing equivocal findings: Some studies showed deteriorations in shooting accuracy with increasing workload (Hoffman et al., 1992; Grebot et al., 2003; Vickers and Williams, 2007; Ihalainen et al., 2018), while other studies indicate no effects (Gallicchio et al., 2016; Luchsinger et al., 2016; Heinrich et al., 2020).

## In a Nutshell: Research on Psychological Factors of Biathlon Peformance

Psychological research in biathlon focused on psychological processes (including neurophysiological mechanisms) underpinning successful biathlon shooting as well as psychological interventions that aim at enhancing performance. Concerning the former, for instance, successful biathlon shooting is related to higher frontal theta power (an electroencephalographic measure), which itself is associated with attentional monitoring processes (Gallicchio et al., 2016; Luchsinger et al., 2016). The importance of focused attention was corroborated by Vickers and Williams (2007) providing evidence that longer final fixations relate to higher shooting accuracies under different psychological (i.e., low vs. high) pressure situations (for contradictory findings, see Heinrich et al., 2020). Also, research based on archival competition data revealed that dealing with pressure may be crucial to successful performance. Lindner (2017) showed that the likelihood of missing the final shot of the final shooting bout turns out to be significantly higher when compared with the previous shots of the final bout, especially in top-ranked biathletes. Furthermore, longer shooting times are often resulting in performance deteriorations (see Lindner, 2017). Additionally, psychological interventions such as autogenic, imagery, or relaxation training combined with specific shooting training tend to enhance shooting accuracy (Laaksonen et al., 2011) and rifle stability (Groslambert et al., 2003). Finally, a recent prospective study showed that dispositional mindfulness (i.e., awareness, refocusing, etc.) might also predict proportions of the variance in shooting performance in advanced biathletes (Josefsson et al., 2020).

## In a Nutshell: Research on Social Context Factors of Biathlon Performance

In contrast to biological and psychological factors, our knowledge about the role of social context for biathlon performance is very limited. Based on archival competition data, Harb-Wu and Krumer (2019) recently examined audience effects by comparing athletes' shooting and skiing performance when competing abroad vs. in their home country (supportive audience). While biathletes with the highest expertise level missed significantly more shots when competing in front of a supportive audience, lower-ranked biathletes did not show performance decrements in shooting but increased skiing performance (i.e., skied faster at home).

## Interim Summary

First, research in biathlon is mainly focusing on biological and psychological determinants in isolation rather than examining these factors in conjunction. Second, the impact of social context has largely been neglected thus far. To ultimately realize a more integrative and interdisciplinary research approach toward biathlon performance under the umbrella of a biopsychosocial framework (see Figure 1), we propose that (at least) three steps need to be taken.

### Step 1: Studying Social Context

As highlighted by the dashed lines surrounding the “social” context in Figure 1, more research addressing the impact of social context on biathlon performance is mandatory. First, the impact of the presence of audience—regardless of the type of audience (see Harb-Wu and Krumer, 2019)—on both skiing and shooting performance has not been examined yet. Second, the only study on social context, thus far, is based on archival competition data; experimental research, however, is lacking. For instance, given that research on social presence has shown that individuals characterized by extraversion and high self-esteem tend to show performance improvements through the presence of others, while individuals characterized by neuroticism and low self-esteem tend to show performance impairments (Graydon and Murphy, 1995; Uziel, 2007), an experimental approach could serve to examine how athletes' personality characteristics interact with the presence vs. absence of an audience in biathlon. Third, if social context matters, then the question arises *if* and *how* the presence vs. absence of direct opponents—be it at the shooting range or on the skiing course—affect biathlon performance. While all biathletes start at the same time in mass start competitions or with a delay based on the result of a previous race (typically sprint) in pursuit, the position on the skiing course always corresponds to an athlete's overall ranking, and competitors are faced with their direct opponents on both the skiing course and the shooting range. It is, hence, conceivable that the number of simultaneously shooting biathletes may affect performance in head-to-head competitions. Finally, social context in the form of familiar vs. unfamiliar environments or cultures may likewise affect competition performance in biathlon. For instance, the next Winter Olympics will take place in China, while Italy will host the Winter Olympics in 2026.

### Step 2: Building Interdisciplinary Bridges

From a biopsychosocial framework perspective, a truly interdisciplinary approach that allows to concomitantly examine biathlon performance from different viewing angles goes beyond looking at relevant factors in isolation. There are initial attempts taking, for example, a biopsychological approach. For instance, studies examining the role of focused attention by means of measuring electroencephalographic activity also considered biological factors by manipulating cardiovascular load immediately before assessing shooting performance. Results on this particular question are somewhat mixed by showing decreased frontal theta power, on the one hand (Gallicchio et al., 2016), or the lack of an effect, on the other hand (Luchsinger et al., 2016). Vickers and Williams (2007) also showed a significant decrease in the duration of the final fixation with increasing physiological workload. By contrast, Heinrich et al. (2020) did not find an effect of physiological workload on fixation durations. Next to biopsychological research, Harb-Wu and Krumer (2019) examined initial biosocial links when showing negative effects of social context on top-ranked biathletes' shooting accuracy as well as positive effects on low-ranked biathletes' skiing performance (representing biological factors). In conclusion, more interdisciplinary approaches are needed to unravel the intricate links between biological, psychological, and social factors determining biathlon performance. Consequently, these approaches offer new opportunities to resolve, for instance, equivocal findings regarding the impact of physiological workload on shooting performance by integrating psychological factors and social context potentially affecting biological factors.

### Step 3: Taking a Big Data Approach

Our final suggestion is to consider taking a big data approach to explain and predict biathlon performance. Big data approaches are still in their infancy as far as the sport sciences are concerned. Such an approach is, for instance, used for tactical analyses in professional soccer (e.g., Rein and Memmert, 2016). Besides characteristics such as featuring a high volume of data that are produced at high velocity, big data are defined by a diverse set of data, and the aim is to capture the entire populations or systems (*n* = all, Kitchin, 2014). Transferred to biathlon, this approach may bring together physiological and biomechanical data from training and competition, psychological factors such as personality characteristics (i.e., traits) or aspects displaying relevant psychological states (e.g., stress and recovery, perceived pressure) as well as social context information at the skiing course and the shooting range (e.g., audience, opponents) in one database. Both external and internal (i.e., individual) factors could be considered by combining multi-methodologically gathered data (e.g., self-reports, tracking data, physiological monitoring, and competition protocols). However, this approach not only requires cross-disciplinary collaborations but also that researchers and practitioners are sensitive to ethical considerations and privacy issues—challenges that are generally associated with big data (Boyd and Crawford, 2012; Spaaij and Thiel, 2017). Despite these challenges, we are convinced that a big data approach is timely and viable to contribute to our understanding of biathlon performance in a truly interdisciplinary and holistic manner as proposed by the biopsychosocial framework.

## Author Contributions

AH conceptualized the study and wrote the original draft. OS did the supervision, wrote, reviewed, and edited the draft. RC-B was also responsible for the conceptualization, supervision, writing, review, and editing of the manuscript. All authors contributed to the article and approved the submitted version.

## Conflict of Interest

The authors declare that the research was conducted in the absence of any commercial or financial relationships that could be construed as a potential conflict of interest.
